# Sterol Glucosyltransferases Tailor Polysaccharide Accumulation in Arabidopsis Seed Coat Epidermal Cells

**DOI:** 10.3390/cells10102546

**Published:** 2021-09-26

**Authors:** Adeline Berger, Marie-Christine Ralet, Elodie Akary, Christine Sallé, Olivier Grandjean, Isabelle Debeaujon, Helen M. North

**Affiliations:** 1 Institut Jean-Pierre Bourgin, INRAE, AgroParisTech, Université Paris-Saclay, 78000 Versailles, France; adeline.berger@inrae.fr (A.B.); elodie.akary@inrae.fr (E.A.); 2 UR1268 BIA, INRAE, 44300 Nantes, France; marie.ralet@inrae.fr

**Keywords:** mucilage, pectin, polysaccharide, seed, steryl glucoside

## Abstract

The conjugation of sterols with a Glc moiety is catalyzed by sterol glucosyltransferases (SGTs). A portion of the resulting steryl glucosides (SG) are then esterified with a long-chain fatty acid to form acyl-SG (ASG). SG and ASG are prevalent components of plant cellular membranes and influence their organization and functional properties. Mutant analysis had previously inferred that two Arabidopsis SGTs, UGT80A2 and UGT80B1/TT15, could have specialized roles in the production of SG in seeds, despite an overlap in their enzymatic activity. Here, we establish new roles for both enzymes in the accumulation of polysaccharides in seed coat epidermal cells (SCEs). The rhamnogalacturonan-I (RG-I) content of the inner layer of seed mucilage was higher in *ugt80A2*, whereas RG-I accumulation was lower in mutants of *UGT80B1*, with double mutant phenotypes indicating that UGT80A2 acts independently from UGT80B1. In contrast, an additive phenotype was observed in double mutants for increased galactoglucomannan (GGM) content. Double mutants also exhibited increased polymer density within the inner mucilage layer. In contrast, cell wall defects were only observed in mutants defective for *UGT80B1*, while more mucilage cellulose was only observed when *UGT80A2* was mutated. The generation of a range of phenotypic effects, simultaneously within a single cell type, demonstrates that the adjustment of the SG and ASG composition of cellular membranes by UGT80A2 and UGT80B1 tailors polysaccharide accumulation in Arabidopsis seeds.

## 1. Introduction

Phytosterols are integral structural components of cell membranes and influence their physicochemical properties and the activity of their associated proteins. A diverse range of sterols are produced in plants, and a proportion of these are derivatized by conjugation of the C3-hydroxyl group to a sugar moiety, most frequently glucose (Glc), to form steryl glucosides (SG) [1]. SG can be further modified by the acylation of the sugar to form acyl steryl glucosides (ASG). SG and ASG are integrated into membranes in a differential manner, and this can contribute to their functional organization through the formation of ordered domains, termed ‘lipid rafts’ [2].

The conjugation of sterols with Glc is mediated by sterol glucosyltransferases (SGTs), and this activity has been demonstrated for two enzymes in Arabidopsis termed UGT80A2 and UGT80B1 [3,4]. The gene encoding *UGT80B1* was found to be allelic to *TRANSPARENT TESTA 15 (TT15),* which when mutated results in lighter colored seed coats due to the defective accumulation of brown flavonoid pigments [5,6]. Mutants in this gene, termed throughout here as *tt15*, also exhibit other seed phenotypes including smaller seeds, abnormal seed morphology, and reduced suberin and cutin in seed coats [3,5]. Moreover, analyses of *ugt80A2* and *tt15 ugt80A2* double mutants indicated that these seed phenotypes are specific to *tt15* mutants. The distinct in vitro substrate preferences, and alterations in mutant seed SG and ASG contents, suggested that, in seeds, UGT80A2 generates the prevalent SG, notably sitosteryl and stigmasteryl glucosides, while UGT80B1 synthesizes a few minor SG and ASG molecules that play key roles in seeds [3]. Nevertheless, stronger decreases in SG and ASG levels were observed in seeds of a double mutant, defective for both genes, indicating a degree of functional redundancy for the glycosylated sterols generated by UGT80A2 and UGT80B1. It was proposed that these SG and ASG molecules could play a role in the trafficking of lipid polyester precursors of suberin and cutin [5].

The accumulation of flavonoid, suberin, and cutin polymers in the seed coat changes its physical and chemical properties, and in so doing, determines its resistance and influences seed physiology traits, such as dormancy and longevity [7]. The seed coat is a maternally-derived tissue that physically separates the embryo and endosperm from the external environment, and the properties of accumulated polymers confer protection against damage by abiotic and biotic factors. On the mature dry seed, the seed coat cells are dead and polymer accumulation occurs prior to the programmed cell death during seed development. In Arabidopsis, the developing seed coat is composed of 5 to 6 superimposed layers, and polymers accumulate differentially between these [8]. The hydrophobic lipid polyesters cutin and suberin appear to be preferentially deposited on the extracellular surface of the most internal and/or external faces of the seed coat, respectively [5,9,10]. The accumulation of condensed tannins occurs early during seed development in the vacuoles of the innermost cell layer, termed the endothelium. These are synthesized as colorless compounds from phenylalanine, via the phenylpropanoid pathway, and become brown when oxidized during seed desiccation [11,12].

In addition to flavonols and lipid polyesters, large amounts of polysaccharides are produced in the two outermost cell layers. These form reinforced cell wall structures in both layers, while the epidermal cells also secrete mucilage polysaccharides into the apoplasm [9,13,14]. The main component of Arabidopsis mucilage is the pectin rhamnogalacturonan I (RG-I) [15]. The polarized deposition of mucilage polysaccharides leads to the formation of a column-shaped cytoplasm, which is then filled with secondary-cell wall material thereby forming a columella, which is linked to the reinforced periclinal and radial cell walls and surrounded by mucilage polysaccharides. The latter are released on imbibition of mature seeds through the fragmentation of the outer primary cell wall. This fragmentation results from localized remodeling during seed development [16], a key factor in the remodeling being the modulation of homogalacturonan (HG) demethylesterification by pectin methylesterase (PME) through PME inhibitors (PMEI) [17]. Following their release, the mucilage polysaccharides form a sticky hydrogel which encompasses the seed. Arabidopsis mucilage comprises a water-extractable outer layer and an inner layer that remains tightly attached to the seed [13,15]. Although *tt15* mutants have previously been reported to have less prominent columella and reduced mucilage sugar contents, in agreement with *UGT80B1:GUS* expression in seed coat epidermal cells (SCEs) [3,5,18], the role for SGTs in polysaccharide accumulation in these cells remained to be fully investigated.

Here, we present a detailed analysis of SCEs phenotypes and demonstrate new roles for both UGT80A2 and UGT80B1 in the distribution and accumulation of polysaccharides in these cells. Specific and common phenotypes were identified in mutants for each of the SGTs indicating that, within a given cell type, the simultaneous adjustment of different SG and ASG pools modulates the functional properties of the membranes. Furthermore, observed phenotype heterogeneity within seed lots and between genetic backgrounds indicated that other genetic factors and environmental parameters influence the activity of UGT80B1, suggesting further complexity in the regulation of SG and ASG composition.

## 2. Materials and Methods

### 2.1. Plant Materials

The mutants *tt15-2* (COB16) [19], *tt15-7* (EAL136), and *ugt80A2-3* (FCH54) (Figure S1) are in the Wassilewskija-4 (Ws-4) accession and were obtained from the INRAE, Versailles T-DNA collection [20], while *tt15-8* (Salk_021175) [21] and *tt15-9* (Salk_103581) [3], from the Salk Institute T-DNA collection, are in the Col-0 accession [22]. The *pmei6-1* (SM_3.19557) mutant is also in the Col-0 accession [17]. Homozygous lines for Ws-4 T-DNA mutants were identified by PCR amplification from genomic DNA extracts using the primers listed in Table S1. The *tt15-2 ugt80A2-3* double mutant was generated by crossing the corresponding single mutants and genotyping F3 plants from F2 seed batches with a *transparent testa* phenotype for the *ugt80A2-3* insertion. Seed production was carried out in a glasshouse (18–28 °C) with a minimum photoperiod of 13 h ensured when required by supplementary lighting. Plants were grown in compost (Treff Substrates, Zwijndrecht, Netherlands; https://jiffygroup.com/, accessed on 21 September 2021) and watered with Plant-Prod nutritive solution (Fertil, Toronto, Ont., Canada; http://www.plantprod.com, accessed on 21 September 2021). As immature seeds can present mucilage defects which are unrelated to the genotype, the seed harvest was carried out by shaking dry plants in paper bags so that only seeds from mature dehiscing siliques were collected. In all the analyses, comparisons were made between seed lots bulked from at least 2 mutant or wild-type plants that had been grown and harvested simultaneously. The number of seeds for a set mass of each genotype was counted using an Elmor C3 counting machine (Elmor, Schwyz, Switzerland, http://www.elmor.com/ accessed on 21 September 2021) for the same seed lots used for mucilage extraction and analysis.

### 2.2. Seed Flotation

Lots of 200 mature dry seeds were prepared in glass phials for each genotype using an Elmor C3 counting machine (Elmor). Seeds were imbibed by gently pipetting 750 µL of water down the side of the phial, and seed flotation was compared after 10 min.

### 2.3. Cytochemical Staining and Immunolabeling of Seed Mucilage

Lots of 100 mature dry seeds were prepared for each genotype using the Elmor C3 counting machine. Mucilage was either stained directly with 0.01% (*w/v*) ruthenium red (Sigma-Aldrich, Saint-Louis, MO, USA, https://www.sigmaaldrich.com/, accessed on 21 September 2021), or after imbibition in 50 mM EDTA pH 8.0, 50 mM HEPES pH 8.0 or 1 M Na_2_CO_3_ for 1 h. After treatment with EDTA, HEPES, Na_2_CO_3_ and CaCl_2_, the seeds were rinsed once in water before staining in ruthenium red for 1 h. Stained seeds were rinsed twice with water and then observed with a stereomicroscope (Axio Zoom, Zeiss, Oberkochen, Germany, http://www.zeiss.fr/ accessed on 21 September 2021) using transmitted light. After imbibition of seeds in water for 16 h, seeds were stained in the same manner with ruthenium red, and then observed with a light microscope (DMRB, Leica Microsystems, Wetzlar, Germany, http://www.leica-meicrosystems.com/ accessed on 21 September 2021). The developing seed sections were obtained from staged siliques that had been embedded in resin (Technovit 7100®, Kulzer, Wehrheim, Germany, https://www.kulzer-technik.com/, accessed on 21 September 2021) and stained with toluidine blue O as described previously [23,24].

Whole seed immuno-labeling of the released adherent mucilage was performed as described previously [15,24] using INRA-RU1 that recognizes RG-I [25], LM21 that binds heteromannan (HM) polymers [26], or JIM5 and JIM7 that bind to either low-ester or high-ester homogalacturonan (HG), respectively [27]. (Plant Probes, Leeds, UK, http://www.plantprobes.net/, accessed on 21 September 2021). These primary monoclonal antibodies were then detected using a rat anti-mouse IgG secondary antibody conjugated to Alexa Fluor 488 (Invitrogen, https://www.thermofisher.com, Waltham, MA, USA, accessed on 21 September 2021). The labeling of cellulose was performed with either 25 µg mL^−1^ Calcofluor or 0.01% (*w/v*) Direct Red 23 (Sigma-Aldrich) in 50 mM NaCl [28]. The seeds and mucilage were observed with a either a Leica SP5 II AOBS Tandem HyD spectral confocal laser scanning microscope (Leica Microsystems), or a Zeiss LSM710 confocal microscope using a 405-nm diode laser line to excite Calcofluor or a 561-nm diode laser line to excite the Direct Red 23, and a 488-nm argon laser line to excite Alexa Fluor 488. The fluorescence emission was detected between 412 and 490 nm, 570 and 650 nm, or 500 and 550 nm for Calcofluor, Direct Red 23, and Alexa Fluor 488, respectively. For comparison of signal intensity within a given experiment, laser gain values were fixed. Mucilage and seed width were measured on images of seeds obtained following double labeling with RU-1 and DR23 as previously described [29].

### 2.4. Scanning Electron Microscopy (SEM) and Measurement of Radial Cell Wall Width

SEM observations were performed as described previously [17], either directly on dry seeds or on seeds that had been imbibed in water for 1 h and then dried on the bench for several days. Measurements of radial cell wall width were carried out using Image J 1.49c (Freeware, National Institutes of Health, Bethesda, MD, USA, http://rsb.info.nih.gov/ij/, accessed on 21 September 2021) with a Cintiq 22HD graphic tablet and pressure sensitive pen (Wacom, Kazo, Japan; http://www.wacom.com/, accessed on 21 September 2021). Thirty imbibed and dried seeds per genotype were analyzed, and six different cells located in the same mid-seed region were measured per seed.

### 2.5. Extraction of Mucilage Polysaccharides

Rates of mucilage release were determined as previously described [17] using 15 mg of dry seeds that were imbibed by mixing in 1.5 mL of distilled water at 20 °C for the indicated times (10, 20, 30, 45, 60, 90, 120, 180, and 300 min), minus 3 min to account for centrifugation time. After imbibition seeds were centrifuged (8000× *g*, 3 min) and 1 mL of supernatant was carefully removed and diluted appropriately with distilled water prior to galacturonic acid (GalA) quantification.

Extracts of outer and inner mucilage layers were obtained from seeds using a sequential extraction procedure. The outer mucilage was extracted by incubating 100 mg of seed with 4 mL of 50 mM Na_2_CO_3_ for 3 h at RT, with continuous head-over-tail mixing. Following centrifugation (8000× *g* for 5 min), the supernatant was carefully removed, stabilized by treating for 5 min at 100 °C, and stored at −18 °C prior to analyses. The seeds were then rinsed three times with 5 mL of water and then three times with 5 mL of sodium acetate buffer pH4.5. Rhamnogalacturonan hydrolase (EC 3.2.1.171, glycoside hydrolase family 28), purified from a technical preparation of *Aspergillus aculeatus,* as previously described [30], was added to washed seeds (0.8 nkat), the volume adjusted to 3 mL, and the reaction incubated for 16 h at 40 °C, as described previously [24]. Following centrifugations (8000× *g*, 5 min), the supernatant was recovered, stabilized, and stored as above for the outer mucilage.

Total mucilage, including cellulose, was extracted using an ultrasonic treatment [31]. Twenty-five mg of seed were incubated with 3 mL of water at RT for 1 h, cooled in an ice bath, and then sonicated for 30 s with a MISONIX S-4000 (30% amplitude). Seeds were then centrifuged (1000× *g*, 1 min) and the supernatant carefully recovered. Seeds were rinsed three times with 1 mL of water and the rinses combined with the supernatant, which was freeze-dried prior to analysis.

### 2.6. Chemical and Physico-Chemical Characterization of Mucilage Extracts

Uronic acid (as GalA) was determined by an automated *m*-hydroxybiphenyl method [32]. For total mucilage extracts obtained using sonication, individual neutral sugars were analyzed by gas-liquid chromatography after being derivatized to alditol acetates [33]. Different acid hydrolysis procedures were applied prior to derivatization: 2 M sulfuric acid at 100 °C for 6 h, or 13 M sulfuric acid at 25 °C for 30 min, followed by 2 M sulfuric acid at 100 °C for 2 h. The former was used to quantify rhamnose (Rha) and non-cellulosic Glc, while the pre-hydrolysis of cellulosic Glc was achieved with the latter. Other sugars, GalA, arabinose (Ara), xylose (Xyl), mannose (Man), and galactose (Gal), were quantified following both procedures.

The macromolecular characteristics of outer mucilage Na_2_CO_3_ extracts were analyzed by high-performance size exclusion chromatography (HP-SEC) coupled to a differential refractometer, a dual laser light scattering, and a differential pressure viscometer as detailed by Saez-Aguayo et al. [34]. Briefly, 1 mL of extract was boiled for 5 min and filtered through a polyvinylidene difluoride filter (13 mm-diameter, 0.45 µm-pore size, Whatman, Maidstone, UK, https://www.cytivalifesciences.com/ accessed on 21 September 2021). HP-SEC analysis was performed at room temperature on a system comprised of a Shodex OH SB-G precolumn, followed by a Shodex OH-Pack SB-805 HQ column. All detectors were calibrated with a pullulan narrow standard (Malvern Instruments, Malvern, UK, https://www.malvernpanalytical.com/ accessed on 21 September 2021) and data analyses were performed using OmniSec version 4.5 software (Malvern Instruments).

### 2.7. Analysis of Fluorescent Probe Permeability and Mobility in Inner Mucilage

For all analyses, seeds were first imbibed for 1 h in water to release mucilage. FITC-dextran staining was then carried out as previously described [35] using 4, 40, 70, and 150-kDa molecules. For the measurement of fluorescence anisotropy, seeds with released mucilage were rinsed twice in phosphate buffer saline (PBS) pH 7.0 and transferred to an eight-well sticky slide (Ibidi, Gräfelfing, Germany; 80828, https://ibidi.com/, accessed on 21 September 2021). Seeds were then incubated in the FITC-polysucrose probe (20 kDa, TdB consultancy, Uppsala, Sweden, https://tdblabs.se/, accessed on 21 September 2021) at 0.1 mg/mL in PBS and incubated for 30 min at RT in the dark. Seeds were observed with an inverted laser-scanning confocal microscope (Zeiss LSM710) equipped with an external BiG module consisting of two GaAsP high-sensitivity detectors, and images were acquired using a 25 × water immersion objective (NA = 0.8), and the standard scanning mode format of 512 pixels × 512 pixels. The Ar 488-nm laser was used to excite FITC, and fluorescence emission was retrieved by a short pass emission filter (610 nm) split into two channels by a polarizing beam splitter cube. The filter in the first channel selected emitted light parallel to the incident light to generate the I_//_ image, whereas the polarizer in the second channel was perpendicular to the incident light and generated the I_⊥_ image. For each experiment, unbound FITC in water (3 µM) was used as an isotropic reference to adjust each detector [36]. The intensities of fluorescence emission through each polarizer were determined for a given region of interest (ROI) in the duplicate images. The ROI was selected in the area of mucilage above the columella. The anisotropy ratio (*r*) was then calculated as previously described [36] using the equation:

*r* = (I_//_ − I_⊥_)/(I_//_ + 2 I_⊥_)

## 3. Results

### 3.1. tt15 Seeds Float Due to Defective Mucilage Release

**Figure 1:**
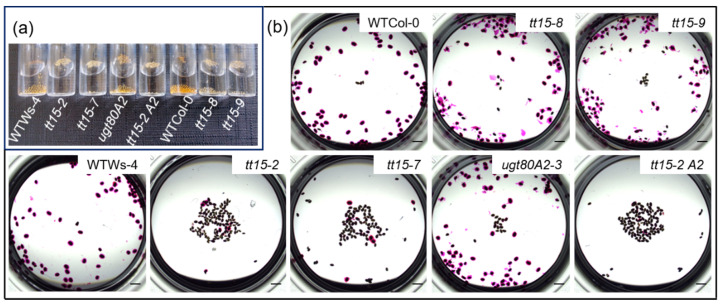
*tt15* seeds remain buoyant because they do not release mucilage. (**a**) Seed buoyancy was examined for 200 seeds of *tt15* or *ugt80A2-3* mutants in Ws-4 or Col-0 accessions by comparing to wild-type seeds after imbibition in water for 10 min. (**b**) direct ruthenium red staining of 100 seeds per genotype. Bars = 1 mm. Wild-type, WT, *tt15-2 ugt80A2-3*, *tt15-2 A2*. Similar results were obtained with seed lots from 3 biological replicates/genotype.

When water is added to wild-type Arabidopsis seeds from Col-0 or WS-4 accessions, most will sink to the bottom of the tube within a few minutes due to an increase in mass resulting from hydrated polysaccharides released from the seed epidermal layer (Figure 1a) [37].

In contrast, when water was added to seeds of *tt15* mutants, the majority floated at the surface (Figure 1a). More seeds sank for *tt15-8* and *tt15-9* seed lots, which are mutant alleles in the Col-0 accession. Furthermore, while seeds affected in *UGT80A2,* the functional paralog of *TT15,* sank like those of wild type, those of a *tt15-2 ugt80A2-3* double mutant floated in a similar manner to *tt15-2*.

The observed flotation of *tt15* seeds was reminiscent of that reported for seeds of natural mutants affected in mucilage release due to mutations in *MUCILAGE MODIFIED2* (*MUM2*), *PRX36*, and *PECTIN METHYLESTERASE INHIBITOR6* (*PMEI6*) [37], or induced mutants in *IRREGULAR XYLEM14* (*IRX14*) and *MUCILAGE-RELATED21* (*MUCI21*) [38]. We therefore examined mucilage release in seeds imbibed in the pectin dye ruthenium red (Figure 1b). After 1 h, a pink halo of mucilage was visible around all the seeds of wild type and the *ugt80A2-3* mutant, and many of those from *tt15* mutant alleles in the Col-0 accession. Relatively few seeds had mucilage halos for *tt15-2*, and *tt15-7* mutant alleles or *tt15-2 ugt80A2-3*. The mutation of *TT15* can thus maintain seed buoyancy through the absence of mucilage release.

### 3.2. UGT80B1 Is Necessary for Fragmentation of the Outer Cell Wall of Seed Coat Epidermal Cells

**Figure 2:**
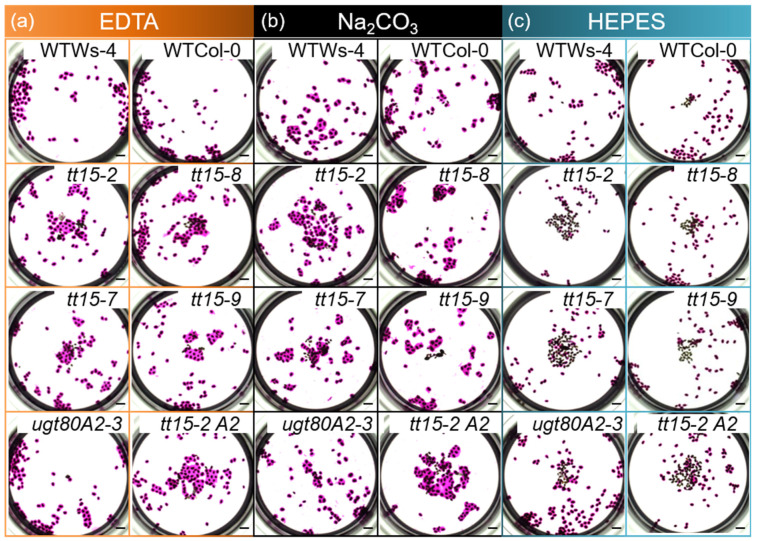
The mucilage release defect in *tt15* mutants is complemented by imbibition in EDTA or Na_2_CO_3_, but not the HEPES buffer. Batches of 100 seeds per genotype were imbibed in (**a**) 50 mM EDTA pH 8.0 (images outlined in orange) (**b**) 1 M Na_2_CO_3_ (images outlined in black) or (**c**) 50 mM HEPES pH 8.0 (images outlined in blue) followed by ruthenium red staining. Wild-type, WT; *tt15-2 ugt80A2-3*, *tt15-2 A2*. Bars = 1 mm. Similar results were obtained with seed batches from 3 biological replicates/genotype.

Seed flotation due to defective mucilage release can result from the modification of the hydrophilic properties of the constituent polysaccharides, or due to defects in the fragmentation of the outer primary cell wall of the SCEs [39]. Mucilage release can be restored in mutants by incubating seeds in the cation chelator EDTA [17,40,41,42] or the weak alkali Na_2_CO_3_ [41]. Nevertheless, forced outer cell wall fragmentation in seeds of *pmei6* differs from wild type with wall material detaching from the surface of cells in large slivers that often become trapped in the adherent mucilage, or are attached to the hilum [17]. To define further the type of mucilage release defect, seeds from *tt15* and *ugt80A2* mutants were imbibed in EDTA or Na_2_CO_3_ before ruthenium red staining. In contrast to imbibition in the control buffer (50 mM HEPES, pH 8.0), mucilage was released from most *tt15* mutant seeds in 50 mM EDTA, pH 8.0 and 1 M Na_2_CO_3_, similar to the wild type (Figure 2). To determine whether the mucilage liberation observed with EDTA was pH-dependent, seeds were also examined when imbibed in EDTA at lower pH. The number of seeds releasing mucilage at both pH 6.0 and 7.0 was similar to those observed at pH 8.0 for all genotypes (Figure S2). The defective mucilage release phenotype of *tt15* mutants can, therefore, be rescued by both cation chelation and alkali pH.

The free carboxyl groups of demethylesterified HG can crosslink polymers through the cation Ca^2+^, forming bridges that reinforce pectin [43], so we examined the effect of exogenous calcium on mucilage release. The difference between *tt15* and wild-type seeds was intensified, and mucilage release was observed in fewer seeds for all genotypes than in the HEPES buffer control (Figure 2 and Figure S3), which was consistent with Ca^2+^ increasing cell wall resistance.

### 3.3. tt15 Seeds Exhibit Delayed and Heterogeneous Mucilage Release

**Figure 3:**
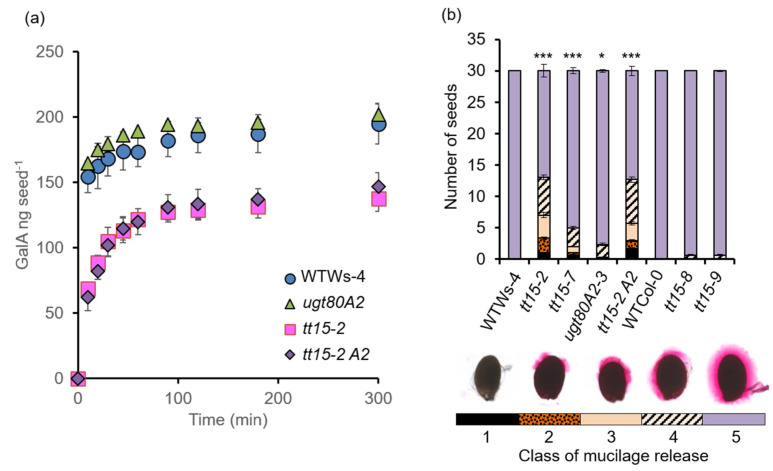
Seed mucilage release is delayed and heterogeneous in *tt15* mutants. (**a**) Rate of mucilage release in water as measured by the amount of released GalA sugars. Error bars represent SD values of 3 biological replicates. (**b**) Quantification of different classes of mucilage release observed on ruthenium red staining of seeds after imbibition in water for 16 h. Five different classes (1 to 5) of mucilage release were assigned according to images of seeds shown with corresponding shading on the graph indicated in bars beneath the images. Error bars represent SE values (*n* = 3). Kruskal-Wallis rank sum test comparing to wild type *** *p* < 0.001, * *p* < 0.05. Similar results were obtained in an independent experiment using biological replicates for each genotype. Wild-type, WT; *tt15-2 ugt80A2-3*, *tt15-2 A2*.

It was noted that more *tt15* seeds released mucilage after 1 h in the HEPES buffer than 10 min in water (Figure 1b and Figure 2). The rate of mucilage release was, therefore, determined by measuring GalA contents in mucilage released into water after incubation for increasing time intervals (Figure 3a), corresponding to mucilage in the outer layer. To adjust for the larger number of *tt15* and *tt15 ugt80A2* seeds in a given mass (Figure S4a, ref. [3,5]), the amount of GalA in mucilage was expressed per seed. The mucilage release rate from *tt15-2* seeds was six times lower than that from wild type seeds (V_max_ [*tt15*] = 13.1 ng min^−1^ per seed vs. V_max_ [wild type] = 77.5 ng min^−1^ per seed), while the V_max_ [*ugt80A2*] was 91.7 ng min^−1^ per seed, similar to wild type (Figure 3a). The V_max_ for the double mutant *tt15-2 ugt80A2* was comparable to *tt15-2* alone (10.4 ng min^−1^ per seed). After 5 h, the rate of GalA release from all genotypes was beginning to plateau, and mutants defective for *TT15* exhibited lower relative GalA contents than the wild type and *ugt80A2*.

**Figure 4:**
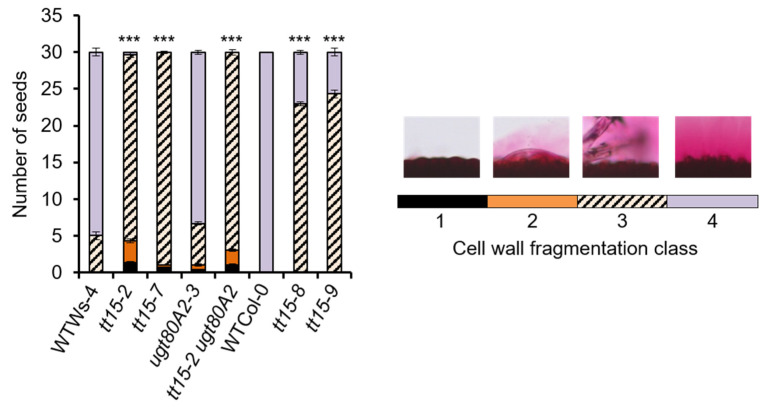
*tt15* mutants are defective for the fragmentation of the outer primary cell wall of seed coat epidermal cells on imbibition. Cell wall fragmentation was quantified in four different classes following ruthenium red staining for 30 seeds/genotype after imbibition in water for 16 h. Classes 1 to 4 were assigned according to the images of the seed surface shown above the corresponding shaded bars used on the graph; in 1, the primary cell wall remained intact, in 2 the primary cell wall was slightly dissociated from the seed surface as blistering, in 3 large slivers of primary cell wall material were observed within the mucilage leaving columella tops naked, and in 4 fragmentation occurred for cells individually, leaving primary cell wall fragments attached to the tops of columella. Error bars represent SE values (*n* = 3). Pearson chi-squared test comparing to wild-type *** *p* < 0.001. Similar results were obtained in an independent experiment using biological replicates for each genotype. Wild-type, WT; *tt15-2 ugt80A2-3, tt15-2 A2*.

Although mucilage release from *tt15* seeds increased with prolonged incubation in water, the halo of mucilage observed around individual seeds was heterogeneous compared to those imbibed in EDTA or Na_2_CO_3_. The extent of mucilage release could be categorized into 5 classes, and the number of seeds in each was determined (Figure 3b). Even after 24 h of imbibition in water, a proportion of *tt15* mutant seeds showed differences in mucilage release compared to the wild type, but this was only significantly different in mutants in the Ws-4 accessions, in particular *tt15-2*. As the mucilage release defect could be rescued in *tt15* with EDTA or Na_2_CO_3_, indicating defective fragmentation of the outer primary cell wall, the extent of cell wall breakage was quantified in the same seeds according to four classes (Figure 4). Seeds of all *tt15* mutant alleles and *tt15-2 ugt80A2-3*, but not *ugt80A2-3,* showed significant differences in wall fragmentation compared to their respective wild type.

### 3.4. HG Methylesterification Is Conserved in Outer Cell Wall Fragments from tt15 Seed Coat Epidermal Cells

**Figure 5:**
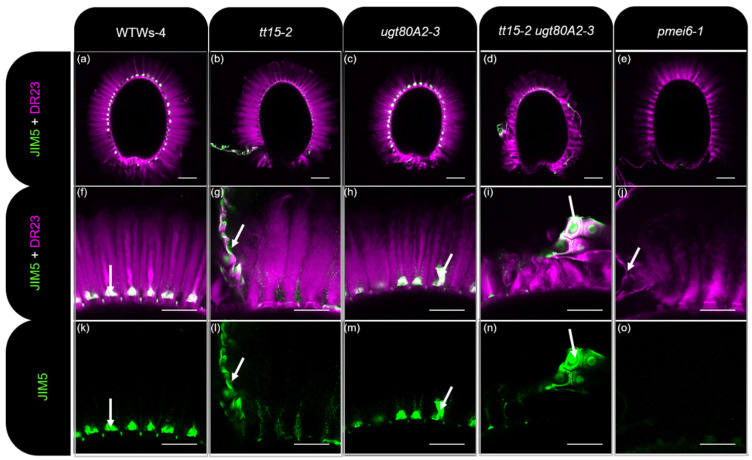
Methylesterified HG is present in outer cell wall fragments released from *tt15* seed coat epidermal cells on imbibition. Confocal microscopy optical sections of seed coat epidermal cells and inner mucilage released from mature seeds after labeling of partially methylesterified HG epitopes with JIM5 antibodies (green) and staining cellulose with Direct Red 23 (DR23; magenta). (**a**–**j**) are composite images of double labeling, whereas (**k**–**o**) are JIM5 labeling alone. (**a**–**e**) show whole seeds, and (**f**–**o**) are higher magnifications of a zone from the same whole seed image above each, respectively. WT, wild type. Bars = 100 µm (**a**–**e**) and 50 µm (**f**–**o**). White arrows indicate outer cell wall fragments within mucilage. All genotypes are in the Ws-4 background, except *pmei6-1* which is in Col-0. JIM5 labelling of outer cell wall fragments in wild type Col-0 [17] resembles that of Ws-4.

Defective outer cell wall fragmentation has previously been associated with higher pectin methylesterase (PME) activity and the loss of methylesterified HG epitopes within *pmei6* mucilage [17]. In contrast, primary cell wall fragments in the mucilage of transgenic seeds overexpressing *PMEI6* exhibited a stronger labeling of methylesterified HG than wild type. To determine whether the cell wall fragmentation defect of *tt15* seeds was due to modified pectin methylesterification, whole mount immunolabeling of seeds and adherent mucilage was carried out using JIM5 and JIM7 monoclonal antibodies to moderately or highly methylesterified HGs, respectively (Figure 5 and Figure S5). As previously documented, while JIM5 and JIM7 labeled cell wall fragments trapped in wild type mucilage, and attached to the tops of columella (Figure 5a,f,k and Figure S5a,b), no labeling was observed for *pmei6* (Figure 5e,j,o and Figure S5k,l [17]). *ugt80A2-3* seeds had equivalent methylesterified HG labeling and cell wall fragments to the wild type (Figure 5c,h,m and Figure S5g,h) in agreement with the absence of a mucilage release phenotype. While the tops of columella were naked in *tt15-2, tt15-7*, and *tt15-2 ugt80A2-3* (Figure 5b,d,g,i,l,n and Figure S5c–f,i,j), as observed for *pmei6-1*, nevertheless, the large pieces of primary cell wall, as well as smaller fragments, were labeled with JIM5 and JIM7. This indicated that defective cell wall breakage in *tt15* mutants was not due to increased HG demethylesterification.

### 3.5. Secondary Thickening of Epidermal Cell Radial Walls Is Increased in tt15 Seeds

**Figure 6:**
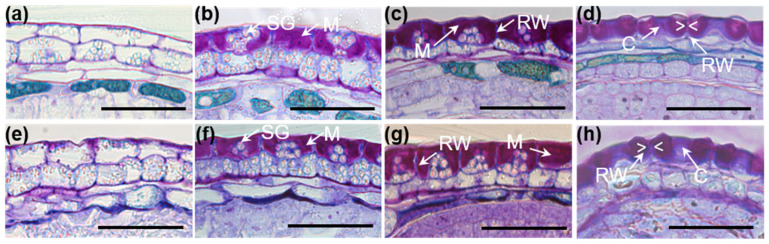
Differentiation of the outer seed coat is delayed in *tt15-2*. Sections of developing seeds were stained with toluidine blue O. (**a**–**d**) wild type Ws-4; (**e**–**h**) *tt15-2.* Seeds at 8 DAP (**a**), 10 DAP (**b**,**e**), 12 DAP (**c**,**f**) 14 DAP (**d**,**g**) and 16 DAP (**h**). C, columella; M, mucilage; RW, radial wall; SG, starch granules. Bars: 50 µm.

The seed coat of *tt15-2* was examined over the key stages of epidermal cell differentiation for changes that could explain the defect in cell wall breakage. Differentiation proceeded through the same steps observed in wild type, except that similar stages in *tt15-2* were observed with a consistent 2-day delay, i.e., 8 days after pollination (DAP) being equivalent to 10 DAP, through to the end of the differentiation process (Figure 6). Furthermore, towards the end of seed coat epidermal cell differentiation, when secondary cell wall reinforcement occurs, the radial cell walls appeared wider than those of the wild type (Figure 6h).

**Figure 7:**
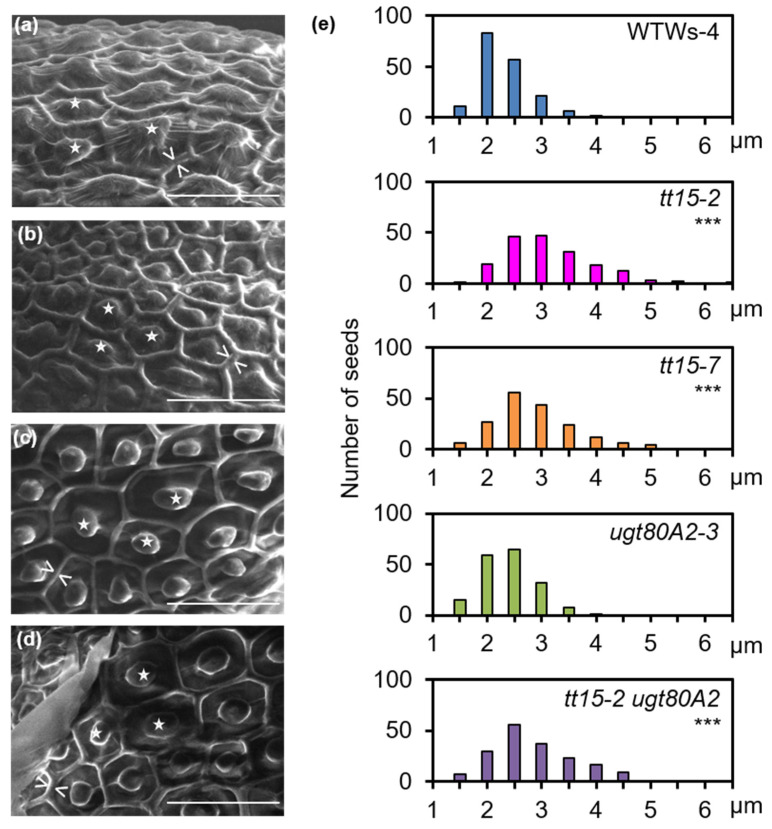
Mutation of *TT15* causes increased thickening of radial walls of seed coat epidermal cells. SEM images of seeds before (**a**,**b**) or after imbibition in water followed by drying (**c**,**d**). (**a**,**c**) wild type Ws-4; (**b**,**d**) *tt15-2*. Asterisks indicate columella and white arrows the outer edges of radial cell walls. Bars: 50 µm. (**e**) Quantification of the width of radial walls of 180 cells from 30 different dry, untreated seeds of each indicated genotype. Kruskal-Wallis test compared to wild type *** *p* < 0.01. *tt15-2 A2*, *tt15-2 ugt80A2.* All genotypes are in the Ws-4 background. Similar results were obtained in an independent experiment using biological replicates for each genotype (Figure S6).

The radial walls of seed coat epidermal cells were examined in more detail by SEM either directly on mature, dry seeds or after imbibition to release mucilage followed by drying. The radial cell walls of *tt15-2* epidermal cells again appeared wider than those of the wild type (Figure 7a–d). The quantification of radial wall width confirmed that they were generally wider in *tt15* mutants and exhibited a larger range of sizes than the wild type (Figure 7e and Figure S6). The radial wall width of *ugt80A2-3* SCEs was not significantly different from the wild type, and there was no apparent additive phenotype in *tt15-2 ugt80A2-3*.

### 3.6. The Accumulation of Seed Mucilage Polysaccharides Is Modified in Both tt15 and ugt80A2 Mutants

**Figure 8:**
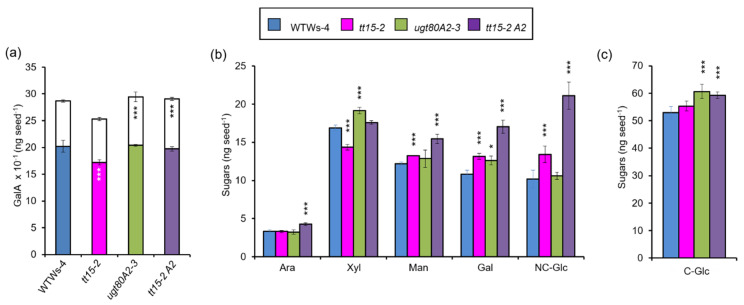
The production of mucilage polysaccharides is modified by mutation of *TT15* and *UGT80A2*. (**a**) Mucilage was extracted sequentially from intact seeds and GalA amounts determined for outer water-extracted mucilage (coloured bars) or RGase hydrolysates of inner mucilage (white bars). Error bars are SE (*n* = 3) for biological replicates. Dunnett pairwise comparison to wild type, *** *p* < 0.01. (**b**) Minor sugar contents. Error bars are SE (*n* = 3–12). (**c**) cellulosic glucose contents in total mucilage extracted by sonication. Error bars are SE (*n* = 6). In (**b**,**c**), significant differences from wild type (Kruskal-Wallis test, * *p* < 0.1, *** *p* < 0.001). Wild type, WT; *tt15-2 ugt80A2-3*, *tt15-2 A2,* NC-Glc, non-cellulose derived glucose, C-Glc, cellulose-derived glucose. All genotypes are in the Ws-4 background.

Similar increases in the radial cell wall width of SCEs have been reported for mutants in the transcription factors *MYB75* and *KNOTTED ARABIDOPSIS THALIANA7* (*KNAT7*) [44]. In the latter, the inner mucilage layer appeared thinner on ruthenium red staining, indicating that polysaccharide production was affected. The lower amounts of outer mucilage quantified for mutants defective for *UGT80B1*, following prolonged imbibition in water (Figure 3a), could be due to the proportion of seeds that had not fully released mucilage (Figure 3b), but could also reflect modified polysaccharide synthesis or partitioning between layers. To specifically assess mucilage polysaccharide synthesis in the sterol glucosyltransferase mutants, the amount of GalA present in each mucilage layer was determined following a sequential extraction procedure that first used Na_2_CO_3_ to rescue the release defect and recover the outer mucilage, followed by RGase treatment to hydrolyze the inner mucilage layer. As above (Figure 3) values were determined per seed to take into consideration that *tt15* and *tt15 ugt80A2* seeds had a lower mass (Figure S4a), all the mutants showed small, but significant alterations in mucilage accumulation (Figure 8a). In agreement with a previous study [18], *tt15* produced fewer total mucilage polysaccharides. Here, we found that this was due to a reduction only in the amounts of outer polymers, and GalA in inner mucilage per seed was proportionally the same as the wild type. In contrast, *ugt80A2* and *tt15 ugt80A2* exhibited no change in total mucilage production per seed, but did have more polysaccharides in the adherent mucilage layer.

**Figure 9:**
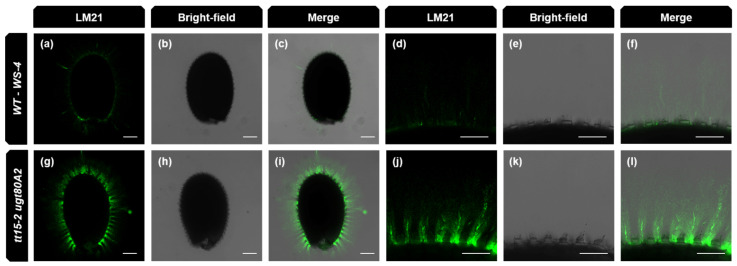
Immunolabeling of inner mucilage heteromannan epitopes is more intense in the double mutant *tt15-2 ugt80A2* than in wild type. Confocal microscopy optical sections of mature seeds and the inner mucilage layer after labeling of heteromannan with LM21 antibodies (green). (**a**,**d**,**g**,**j**) show LM21 labeling alone, (**b**,**e**,**h**,**k**) are bright-field images and (**c**,**f**,**i**,**l**) are composite image of labeling and bright-field. (**a**–**c**) and (**g**–**j**) show whole seeds, and (**d**–**f**) and (**j**–**l**) are higher magnifications of a zone from the corresponding whole seed image. WT, wild type. Bars = 100 µm (**a**–**c**) and (**g**–**j**) and 50 µm (**d**–**f**) and (**j**–**l**).

Minor sugar amounts and cellulose-derived Glc were also determined following the extraction of total mucilage using sonication [31]. This method recovers all mucilage polymers in both layers, including cellulose microfibrils. The GalA contents for total mucilage from *tt15* were again lower than the wild type, with similar reductions observed for Rha (Figure S7). This was expected, as the major constituent of mucilage is RG-I and its backbone is formed of a repeating [→2)-α-L-Rha*p*-(1→4)-α-D-Gal*p*A-(1→] disaccharide unit. Xyl contents also had similar reductions in *tt15* (Figure 8b) in agreement with the tight proportionality observed for this sugar in relation to Rha and GalA, and the proposed presence of xylan sidechains on RG-I added through a multi-glycosyltransferase complex [45,46]. Man, Gal and non-cellulosic Glc also showed significant increases in *tt15* mutants that were highest in *tt15 ugt80A2* indicating an additive effect of *UGT80A2* mutation. Concomitant changes in the amounts of these three sugars have previously been observed for *cellulose synthase-like A2* (*csla2*), and *muci10* mutants defective for the synthesis of the hemicellulose galactoglucomannan (GGM), which is a component of inner mucilage [35,47]. To determine whether GGM production was modified, adherent mucilage was labeled with the LM21 antibody that recognizes HM epitopes. Differences in the intensity of labeling within adherent mucilage compared to the wild type were apparent for *tt15 ugt80A2* (Figure 9).

Interestingly, the production of mucilage cellulose was affected in the *ugt80A2* and *tt15 ugt80A2* mutants, with similar increases of around 15 or 12%, respectively (Figure 8c), indicating that there was no additive effect and this resulted uniquely from *UGT80A2* mutation. These modest increases did not result in visible differences in the staining intensity observed with the cellulose specific-dye Direct Red 23 (DR23) (Figure S8).

### 3.7. The Density of the Inner Mucilage Polysaccharide Network Is Altered by Mutation of Sterol Glucosyl Transferases

**Figure 10:**
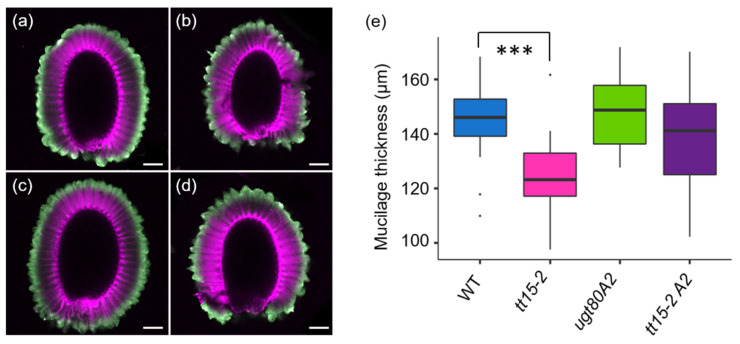
The width of the inner mucilage layer is reduced for *tt15* single mutant seeds. (**a**–**d)**, composite images of confocal microscopy optical sections of seeds immuno-labeled with an antibody against rhamnogalacturonan I (green) to delimit the outer edge of the inner mucilage layer and stained with Direct Red 23 (magenta) to label cellulose in the mucilage and at the seed surface. (**a**), wild type; (**b**), *tt15-2*; (**c**), *ugt80A2*; (**d**), *tt15-2 ugt80A2*. Scale bars: 100 µm. (**e**), Measurements of mucilage width for each genotype. Dunnett pairwise comparison test to wild type *** *p* < 0.01%, *n*= 30. WT, wild type; *tt15-2 A2*, *tt15-2 ugt80A2.* All genotypes are in the Ws-4 background.

The width of the inner mucilage layer was also determined for *tt15* and *ugt80A2* mutants using a double-labeling procedure [29]. This highlights the periphery of the inner mucilage layer through INRA-RU1 antibody labelling, and stains cellulose containing structures on the seed surface with DR23 (Figure 10a–d). Despite the inner mucilage of *ugt80A2* and *tt15 ugt80A2* containing more polysaccharide, there was no proportional increase in the width of the inner layer compared to the wild type (Figure 9e). In contrast, the inner mucilage layer of *tt15* seeds was thinner than the wild type, despite having equivalent amounts of polysaccharides. In all the mutants, therefore, mucilage polysaccharides must be more densely packed. Seed width was also determined from images and confirmed that the differences in seed weight between genotypes (Figure S4a) was indeed related to seed volume (Figure S4b).

The density of gels is governed by polymer composition and properties. An analysis of the macromolecular properties of the outer mucilage extracts, which are almost uniquely composed of unbranched RG-I [15], indicated that the size and conformation of these polymers was not altered in *tt15* and *ugt80A2* mutants compared to the wild type (Table S2). The values obtained for the Ws-4 accession did, however, differ markedly from previously published values for the Col-0 accession [15,45,48]; notably, the major population of polymers were larger and occupied a smaller volume. The properties of the inner mucilage polymers cannot be determined in the same manner because the extraction processes used hydrolyze to shear the polymers; nonetheless, the porosity of seed mucilage networks can be examined in situ using dextran molecules labelled with fluorescein isothiocyanate (FITC) [35,49]. A range of dextran sizes were tested to examine the inner mucilage density in *tt15* and *ugt80A* single and double mutants (Table S3), but no clear differences could be distinguished in the images. As an alternative, the analysis of fluorescence anisotropy was employed to analyze the mucilage of the double mutant. This technique measures the rotational dynamics of molecules in a solution, which is dependent on the solution viscosity as well as the size and shape of the rotating molecule and, in the absence of artefacts, anisotropy is independent of fluorophore concentration. The anisotropy of a 20-kDa FITC-polysucrose probe within the inner mucilage was calculated from ratiometric fluorescence intensities measured using polarized light and comparing values obtained with the polarizer either parallel, or perpendicular, to the polarized excitation. Calculated values are equal to zero in the case of free rotation and increase with the viscosity of the medium surrounding the probe. The anisotropy values measured for the 20-kDa fluorescent probe in the double mutant *tt15 ugt80A2* were significantly higher than those of the wild type, (Table 1) in accordance with their mucilage being formed of a denser polysaccharide network.

## 4. Discussion

While sterols and their derivatives play key structural roles in plant membranes, the precise role of the abundant conjugated sterols, SG and ASG, remains to be established. The glucosylation of sterols in seeds is catalyzed by two SGTs, UGT80A2 and UGT80B1, and mutant seed SG profiles indicate that UGT80A2 catalyzes the production of major SG, and UGT80B1 minor SG and ASG compounds [3]. This might appear counter-intuitive as most, if not all, of the documented phenotypes for mutant seeds have, to date, been attributed to the mutation of *UGT80B1*—notably the accumulation of flavonoid and lipid polyester polymers in the seed coat [3,5,6]. The present study has extended the roles for conjugated sterols in polymer accumulation in seeds for both UGT80A2 and UGT80B1 enzymes through the demonstration of additional mutant phenotypes related to polysaccharide deposition in SCEs.

### 4.1. UGT80A2 and UGT80B1 Sterol Glucosyltransferases Influence the Accumulation of Mucilage Polysaccharides

The accumulation of mucilage polysaccharides was modified in both *tt15* and *ugt80A2* mutants; however, the changes observed indicated distinct roles for the products of each of the SGTs in this process. Firstly, minor changes in the RG-I amount were observed, with *tt15* mutants having less RG-I in the outer mucilage layer, while *ugt80A2* mutants had more inner mucilage RG-I (Figure 8a and Figure S7). This suggests that the specific SG and ASG populations generated by each of the SGTs make unique contributions to mucilage RG-I accumulation. A second specificity was indicated for UGT80A2 in cellulose production with more cellulose in *ugt80A2* inner mucilage, while no change was observed for *tt15* compared to the wild type (Figure 8c). Interestingly, the *tt15 UGT80A2* mutant exhibited equivalent increases in inner mucilage sugars for RG-I and cellulose to those observed for *ugt80A2* alone, while the reduced amount of outer mucilage RG-I in *tt15* was not observed in the double mutant (Figure 8a,c). This suggests that SG produced by UGT80A2 fulfill a role in synthesis or secretion processes that lead to inner mucilage RG-I and cellulose accumulation, and that this is independent of the function of SG and ASG generated by UGT80B1. Mucilage cellulose has been shown to play a critical role as a scaffold for the attachment of RG-I polymers for the formation of the inner mucilage layer [24,48,50,51]. This means that the increased amount of cellulose in *ugt80A2* and *tt15 ugt80A2* (Figure 8c) could drive the concomitant increases in inner layer RG-I (Figure 8a), and that UGT80A2-generated SG may play a direct role on cellulose production alone.

In contrast, deficiency for both UGT80A2 and UGT80B1 activities yielded greater increases in the sugars that are constituents of GGM compared to single mutants alone (Figure 8b). The additive phenotype in the double mutant thus suggests that the SG produced by each enzyme contribute independently to the same cellular function leading to GGM accumulation. This concurs with SG and ASG produced by UGT80A2 having a specific role in RG-I and cellulose production. Together, these observations suggest that the range of SG and ASG produced by UGT80A2 and UGT80B1 provide specific membrane properties that can be tailored for different, or overlapping, functions occurring in parallel within a given cell. This partial functional redundancy contrasts with the overlapping, but not completely redundant functions described for tomato SGTs in different organs [52], which suggests that a higher degree of functional specialization can occur within a given cell or organ.

Sitosterol-β-glucoside was previously proposed to play a role as a primer for cellulose synthesis based on products obtained from in vitro activity assays using cotton fiber membranes [53]. If SGTs play this key role in the production of primers for cellulose synthesis, their corresponding mutants would be expected to be cellulose-deficient, which contrasts with the increased amounts of cellulose observed in *ugt80A2* mucilage, or the unchanged levels observed for *tt15* (Figure 8c). Our observations are in accordance with those of Debolt et al. [5], who also found no reduction in cellulose in whole seeds and vegetative tissues of the double mutant. This led them to conclude that if SG act as primers for cellulose synthesis, the trace amounts of residual SG in double mutant seeds must be sufficient to fulfil this function.

### 4.2. Steryl Glucosides Produced by UGT80A2 and UGT80B1 Modulate the Density of the Inner Mucilage Polymer Network

The volume of the inner layer of seed mucilage is determined not only by the quantity of polymers from which it is formed, but also their macromolecular and physicochemical properties, which dictate conformation and interactions, respectively. Changes in the width or quantity of mucilage polymers in *ugt80A2* and *tt15* mutants indicated their inner mucilage contained more polymers for a given volume than wild type and were more densely packed (Figure 3a and Figure 10). This was confirmed for the double mutant *ugt80A2 tt15-2* by the reduced mobility of a fluorescent-polysucrose probe within the inner mucilage (Table 1). Increased mucilage density has previously been reported for the inner mucilage of *csla2* and *muci10* mutants deficient for GGM production, and in *mum2* mutants defective for Gal side-branch trimming from RG-I [23,35,46,47]. The constituents of GGM and HM labeling were increased in *tt15 ugt80A2* (Figure 3b), and so opposite effects on density would be expected to those of *csla2* and *muci10*, meaning that the change in GGM amount is unlikely to explain the increased density in double mutants. In contrast, polymers in the inner layer of *mum2* mucilage were shown to be packed very densely with more polymers than the wild type, forming a very thin inner mucilage layer [23]. The Gal branches on RG-I are oxidized, probably by a putative Gal oxidase called RUBY, and can form crosslinks to other sugars [54], and relatively small increases in Gal branching (Gal/100 Rha ratio of 7.2 in *mum2* vs. 2.0 in the wild type) have a strong effect on polymer density [23]. This means that the observed increase in *tt15 ugt80A2* polymer density could result from a trivial proportion of the higher Gal levels being derived from increased Gal side-branches on RG-I, and could also be sufficient to counter the effects of higher GGM on mucilage polymer packing. Unfortunately, this hypothesis cannot be validated, as such small changes in RG-I branching are outside the detection limits of current biochemical and cytological methods.

### 4.3. Differences in Seed Size Can Mask Mutation Effects on Seed Constituents

The characterization of embryonic development previously found that *tt15 ugt80A2* mutant embryos exhibit elongation defects from the late heart-stage onwards [5]. Our results show that seed coat development is also affected, with a 2-day developmental delay (Figure 6). The defective elongation of embryos likely explains, at least in part, the reduced seed size and mass observed for *tt15* mutants (Figure S4). In contrast to previous studies [3,5], we did not observe the small reduction in *ugt80A2* seed mass, suggesting plasticity for this phenotype due to environmental differences in cultivation or harvest, genetic differences between alleles or the Ws-2 and Ws-4 backgrounds used previously and here, respectively. The importance of accounting for seed size differences when comparing mucilage composition is demonstrated by comparing the profiles of mucilage release expressed per mg of seed (Figure S9) to those expressed per seed (Figure 3a). While the difference in rate of release between *tt15* mutants and the wild type is evident when using both methods of calculation, all four genotypes reached a similar plateau for total GalA release when compared to a fixed weight of seed, whereas lower mucilage release was apparent for *tt15* mutants when expressed per seed. Only the latter was coherent with the defect in the mucilage release observed for *tt15* mutants after 16 h of imbibition (Figure 3b). When the seed size was not considered, defects in mucilage sugar amounts were imperceptible, and this demonstrates the importance of accounting for this factor in the analysis of mucilage sugar contents.

### 4.4. SG and ASG Synthesized by UGT80B1 Impact Cell Wall Components in Seed Coat Epidermal Cells

Additional phenotypes were observed in *tt15* mutants that indicated other modifications to polysaccharides in SCEs. Firstly, the accumulation of secondary cell wall material was altered as radial cell walls were on average thicker in both *tt15* and *tt15 ugt80A2* (Figure 6 and Figure 7) indicating a localized increase in cell wall polymer deposition. A similar thickening of radial cell walls has been observed in mutants of transcription factors that control the biosynthesis of secondary cell wall polymers, including cellulose and hemicellulose [44,55]. Also, modifications to membrane sterol profiles has previously been shown to affect the homeostasis of cell wall components [56]. Secondly, delayed mucilage release in *tt15* mutants was accompanied by defective fragmentation of the outer primary cell wall (Figure 3a,Figure 4, Figure 5 and Figure S5). In wild-type seeds, the primary cell wall in the zone above the radial cell wall undergoes local remodeling by the peroxidase PRX36 leading to wall fragmentation at this weakened zone under the pressure of swelling mucilage polysaccharides [16]. The targeting of PRX36 to this cell wall zone requires the inhibition of PME activity by the proteinaceous inhibitor PMEI6, so that HG remain methylesterified in this zone of the cell wall. Seeds of both *prx36* and *pmei6* have defective cell wall fragmentation and mucilage release [17,41]. As a result of unregulated PME activity in *pmei6*, the fragments of the primary cell wall observed following mucilage release are not labelled with antibodies to methylesterified HGs (Figure 5e,j,o and Figure S5k,l [17]). While the defective primary cell wall fragmentation observed for seeds of *tt15* mutants was similar to that of *pmei6* seeds, this was not due to uncontrolled demethylesterification, as labelling of methylesterified HG epitopes was observed, which was particularly notable in the large fragments of the primary cell wall (Figure 5g,h,l,n and Figure S5d,f). UGT80B1 could, therefore, have a direct effect on the localization or activity of PRX36, or the target it remodels, in the cell wall. Nonetheless, the relative dynamics of mucilage release determined here for *tt15* mutants, compared to the wild type, were different to those previously reported for *pmei6* seeds (Figure 3a, ref. [17]). Here, *tt15* seeds released mucilage five times slower than the wild type and reached a plateau after 6 h of imbibition (Figure 3a), whereas the rate of mucilage release from *pmei6* seeds was only 2–3 × slower than the wild type, but continued to rise for the following 24 h. The defect in *tt15* SCEs cell wall fragmentation could, therefore, involve a different component of the primary cell wall. To gain further insight into how the SG and ASG conjugated by UGT80B1 contribute to primary cell wall fragmentation, it will be interesting to study whether PRX36 localization is affected in *tt15* mutants.

### 4.5. Mucilage Release Phenotypes Caused by TT15 Mutation Exhibit Variable Penetrance and Expressivity

The level of mucilage release and primary cell wall fragmentation were found to vary between *tt15* mutant seeds from the same mother plant, and also from different genetic backgrounds (Figure 1, Figure 2, Figure 3b and Figure 4). The phenotypes of the T-DNA insertion mutant alleles in Col-0 had milder phenotypes than those in Ws-4. This highlighted a difference in the expressivity of these phenotypes between seeds and variation in penetrance according to background. Weaker phenotype penetrance in Columbia also explains why *tt15-1* seeds were previously reported to produce a mucilage layer to the same extent as the wild type, as this allele is in the Col-2 background [6]. Moreover, *tt15-1* is expected to be a strong mutant allele as it contains a point mutation that introduces a premature stop codon that is likely to cause a complete loss of UGT80B1 function [57]. Variation in phenotype expressivity and incomplete penetrance indicate that another locus, or loci, is polymorphic between these accessions and acts as a genetic modifier of *UGT80B1,* and that this and *UGT80B1* could be affected by environmental parameters.

## 5. Conclusions

The new seed phenotypes presented here for *tt15* and *ugt80A2* mutants show that SG and ASG synthesized by UGT80A2 and UGT80B1 tailor the composition and distribution of polysaccharides in the epidermal cell layer of the seed coat and act in a partially redundant manner. The SG and ASG in SCEs membranes could affect polysaccharide production and/or their secretion to the apoplasm. Notably, membrane rafts containing SG and ASG could influence the localization or function of enzymes involved in the production of mucilage polysaccharides; pectin and GGM are synthesized by glycosyltransferases bound to the membranes of the Golgi apparatus, while cellulose synthase complexes are localized in plasma membranes. Alternatively, the rafts could modulate the efficiency of polymer transport to the apoplasm. Mucilage production and/or accumulation has already been shown to require protein complexes that control trafficking by the trans-Golgi network, or polarized vesicle exocytosis [58,59], and SG- and ASG-containing membrane rafts could contribute to their formation or stability, similar to the role previously proposed for UGT80A2 and UGT80B1 in the trafficking of the polyester precursors for suberin and cutin [5]. Collectively, the seed coat phenotypes observed for *tt15* and *ugt80A2* reinforce the premise that they play partially overlapping functions through the formation of particular SG, ASG, or subpopulations of conjugated sterols having specialized membrane functions, such as raft formation.

## Figures and Tables

**Table 1:** Comparison of fluorescence anisotropy for a 20-kDa FITC-polysucrose probe in seed mucilage of the *tt15 UGT80A2* mutant with wild-type. Values are arbitrary units with SE values in parentheses (*n* > 29) from 15 to 20 different seeds. Asterisks indicate a significant difference from wild type (non-parametric nparcomp multiple comparison test) *** *p* < 0.001, * *p* < 0.05).

Genotype	Biological Replicate 1	Biological Replicate 2
Wild type Ws-4 ^1^	0.0306 (±0.0003)	0.0228 (±0.0002)
*tt15-2 ugt80A2-3* ^1^	0.0329 (±0.0003) ***	0.0291 (±0.0006) *
*tt15-2 ugt80A2-3* ^2^	0.0341 (±0.0005) ***	0.0240 (±0.0003) ***

The columella in mucilage region analyzed had either: ^1^ Primary cell wall fragments attached to their summits. ^2^ ‘Naked’ summits without cell wall fragments.

## Supplementary Materials

The following are available online at https://www.mdpi.com/article/10.3390/cells10102546/s1, Figure S1: Schematic representation of the structure of *UGT80A2* and *UGT80B1* genes indicating the sites and orientation of T-DNA insertions. Figure S2: Complementation of the *tt15* mucilage release defect by EDTA is independent of pH; Figure S3: Effect of Ca^2+^ on the *tt15* mucilage release defect; Figure S4: Mutation of *TT15* reduces seed size; Figure S5: Highly methylesterified HG is present in outer cell wall fragments released from *tt15* seed coat epidermal cells on imbibition; Figure S6: Quantification of radial cell wall width of seed coat epidermal cells on dry, untreated seeds for a biological replicate of Figure 7e. Figure S7: Levels of rhamnogalacturonan I sugars in total mucilage extracted by sonication; Figure S8: The inner mucilage cellulose of *tt15-2 ugt80A2* seeds has a similar staining intensity with the cellulose specific dye Direct Red 23 to that of wild type (WT) WS-4. Figure S9: Rate of mucilage release in water expressed as the amount of GalA sugars for a given mass of seed; Table S1: Sequences of primers used for genotyping T-DNA insertion for mutant alleles in Ws-4 background; Table S2: Macromolecular characteristics of outer mucilage extracted with water from seeds of wild type, *tt15-2, ugt80A2-3* and *tt15-2 ugt80A2-3*; and Table S3: Summary of FITC-dextran molecules used to examine inner mucilage porosity in different genotypes.
