# Supplementary material for: Sterol Glucosyltransferases Tailor Polysaccharide Accumulation in Arabidopsis Seed Coat Epidermal Cells
Source: Cells. 2021 Sep 26;10(10):2546. doi: 10.3390/cells10102546 (PMC8533880; doi:10.3390/cells10102546)
Supplement: Supplementary file 1 [file cells-10-02546-s001.zip › cells-1297203-supplementary.pdf]

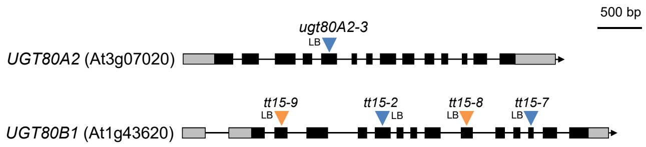

Figure S1. Schematic representation of the structure of *UGT80A2* and *UGT80B1* genes indicating the sites and orientation of T-DNA insertions. Bars indicate the position of exons and are filled in black for the coding-sequence with untranslated regions in grey. Blue triangles indicate alleles in the Ws-4 background and orange triangles alleles in Col-0; LB, left border.

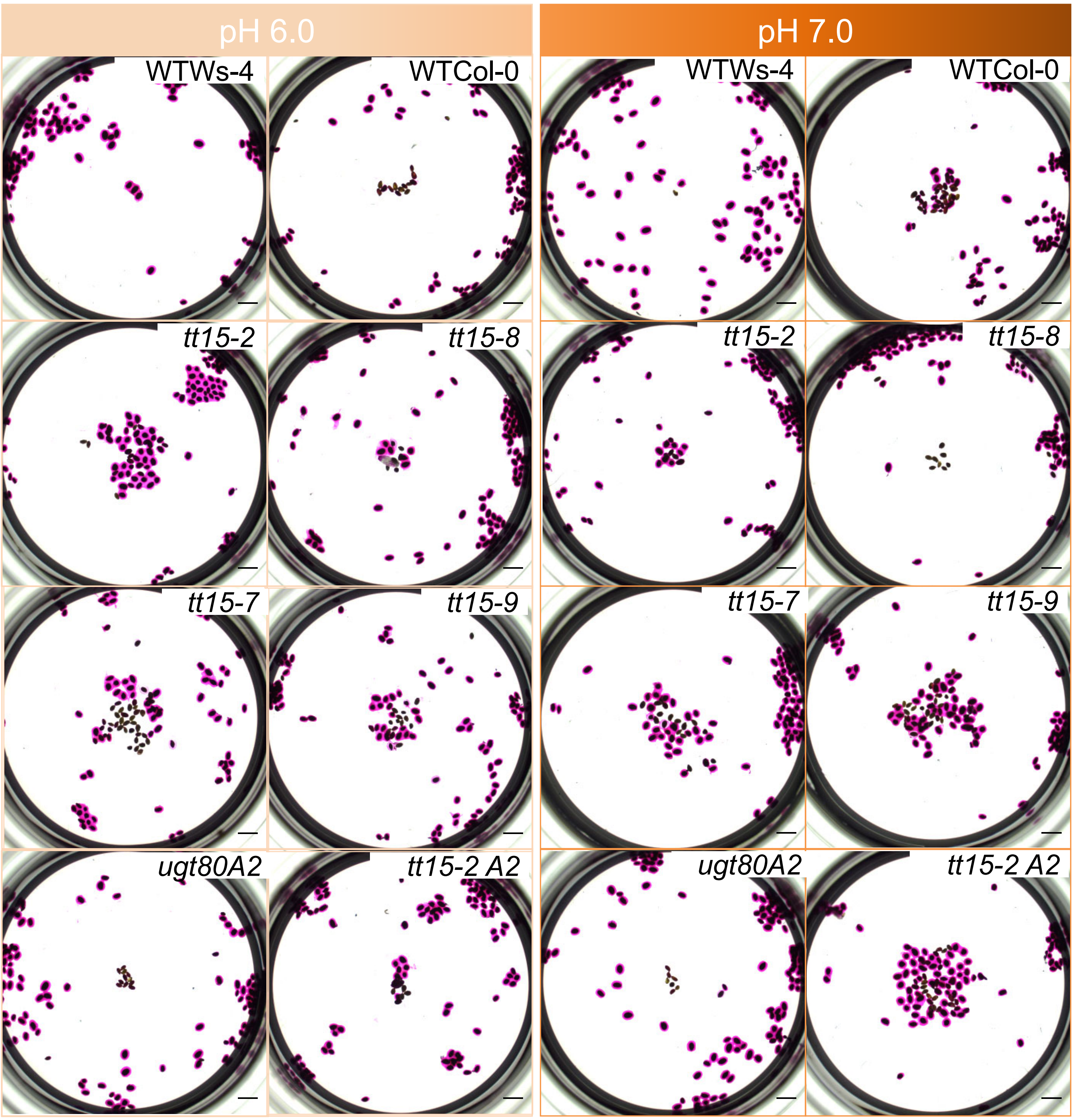

Figure S2. Complementation of the *tt15* mucilage release defect by EDTA is independent of pH. Batches of 100 seeds per genotype were imbibed in 50 mM EDTA at pH 6.0 (images outlined in pale orange) or pH 7.0 (images outlined in dark orange) followed by ruthenium red staining. Wild-type, WT; *tt15-2 ugt80A2-3*, *tt15-2 A2*. Bars = 1 mm. Similar results were obtained with seed batches from 3 biological replicates/genotype.

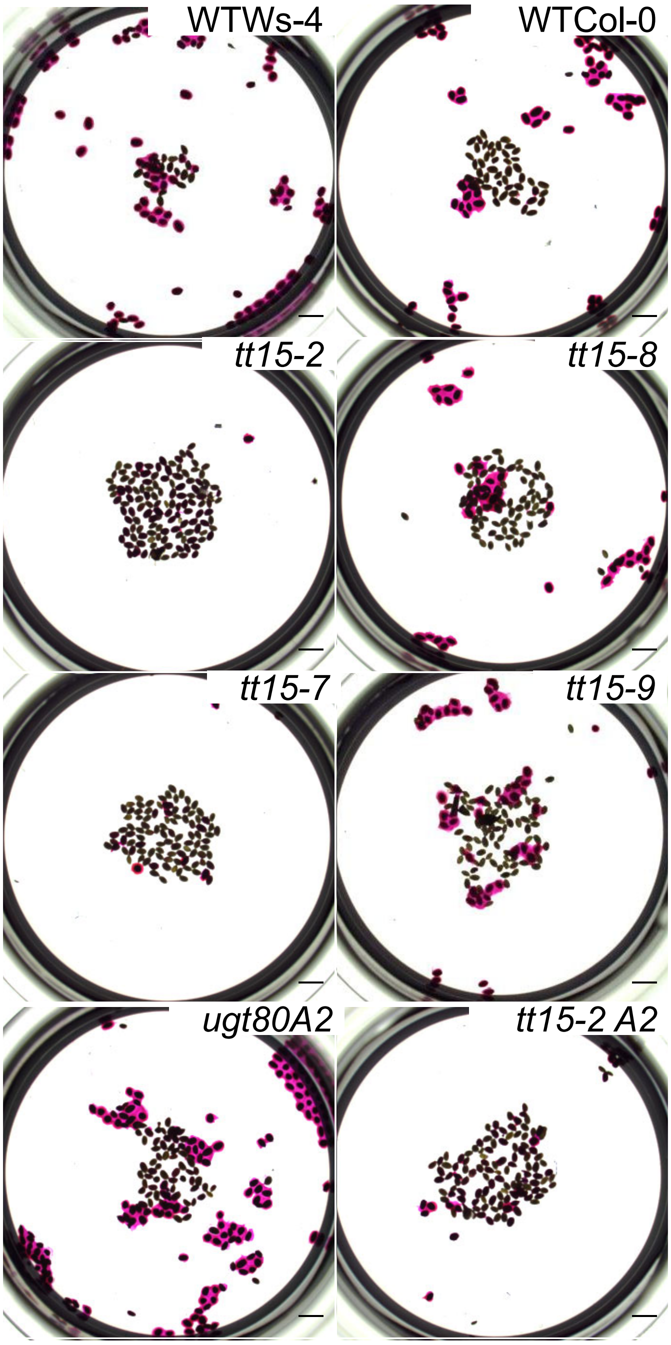

Figure S3. Effect of Ca^{2+} on the *tt15* mucilage release defect. Seeds lots of 100 seeds per genotype were imbibed in 50mM $CaCl_2$ and then stained with ruthenium red Wild type, WT; *tt15-2 ugt80A2-3*, *tt15-2 A2*. Bars = 1 mm.

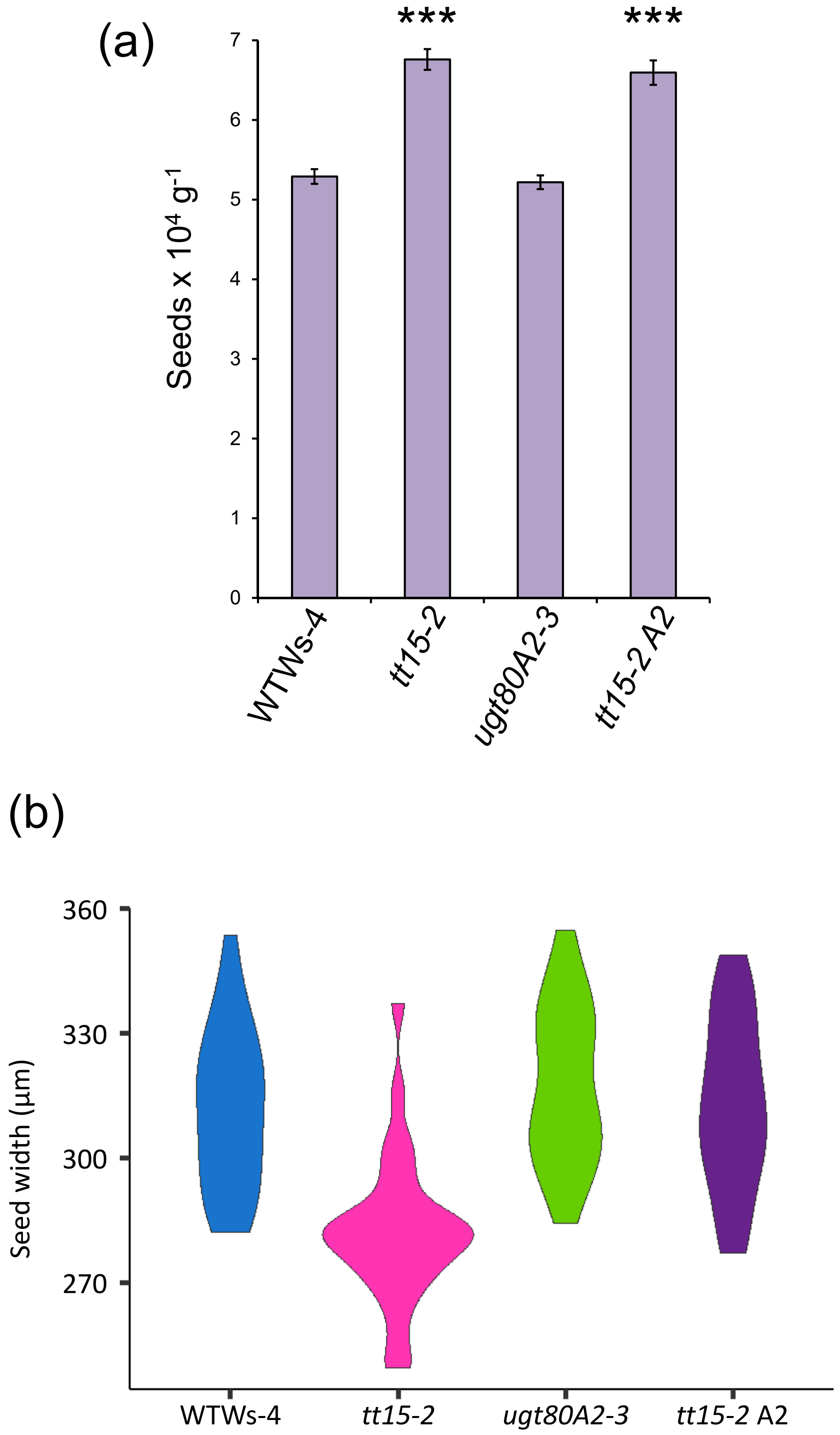

Figure S4. Mutation of *TT15* reduces seed size. (a) Number of seeds observed for a given weight. Error bars are SE (n=3) for biological replicates. Dunnett pairwise comparison to wild type, $P < 0.01$, ***. (b) Patterns of seed width distribution for seed lots. Wild type, WT; *tt15-2 ugt80A2-3*, *tt15-2 A2*.

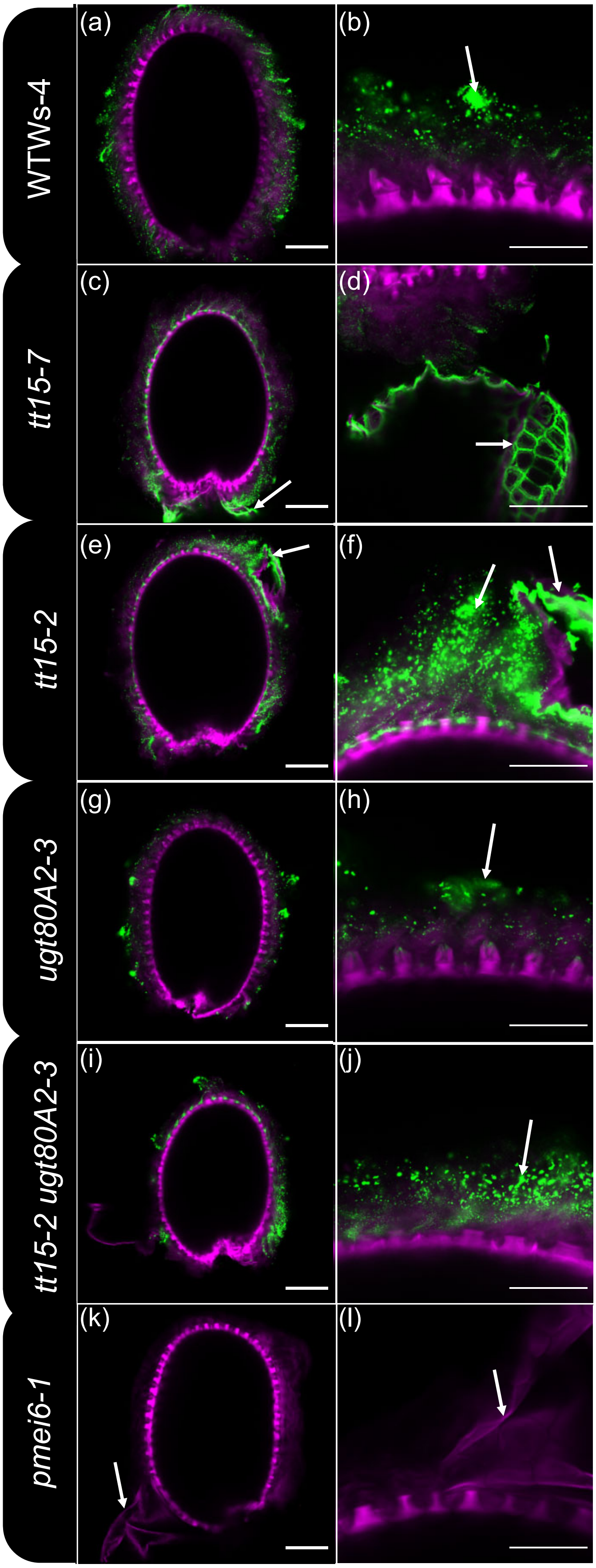

Figure S5. Highly methylesterified HG is present in outer cell wall fragments released from *tt15* seed coat epidermal cells on imbibition. Confocal microscopy optical sections of seed coat epidermal cells and adherent mucilage released from mature seeds showing composite images of labeling of highly methylesterified HG epitopes with JIM7 antibodies (green) and staining of β-glucans with Calcofluor (magenta). (a, c, e, g, i and k) show whole seeds, and (b, d, f, h, j and l) higher magnifications of a zone from the corresponding whole seed image. WT, wild type. Bars = 100 (a, c, e, g, i and k) and 50 µm (b, d, f, h, j and l). White arrows indicate outer cell wall fragments within mucilage.

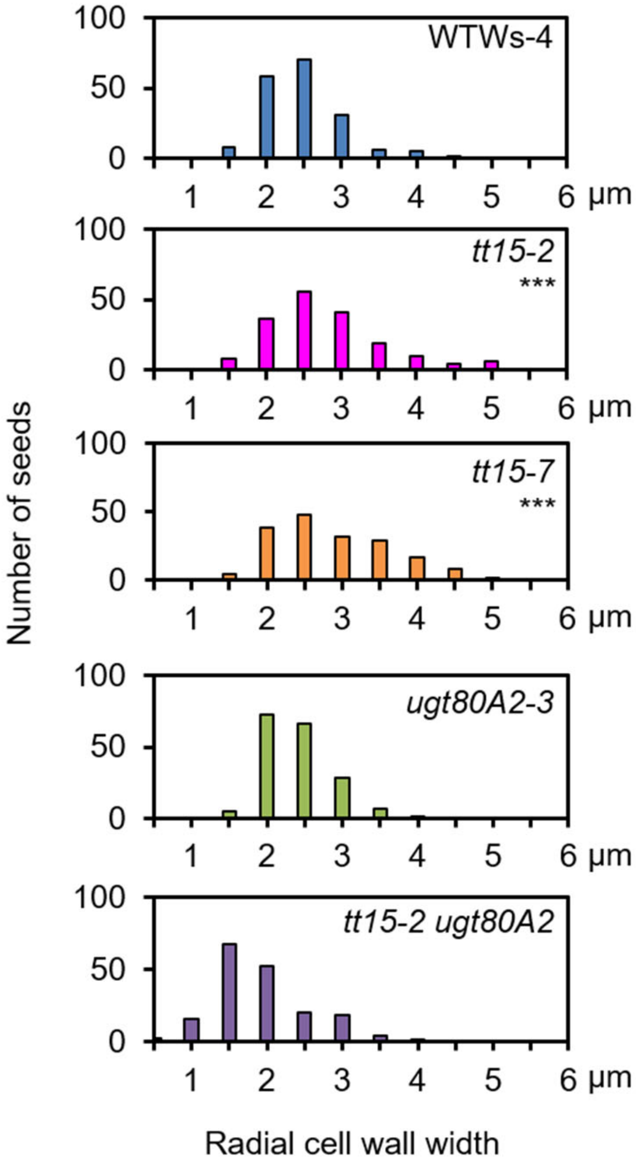

Figure S6. Quantification of radial cell wall width of seed coat epidermal cells on dry, untreated seeds for a biological replicate of Figure 7e. The width of radial walls of 180 cells from 30 different seeds of each indicated genotype were measured. Kruskal-Wallis test compared to wild type $P < 0.01$, ***. *tt15-2 A2*, *tt15-2 ugt80A2*. All genotypes are in the Ws-4 background.

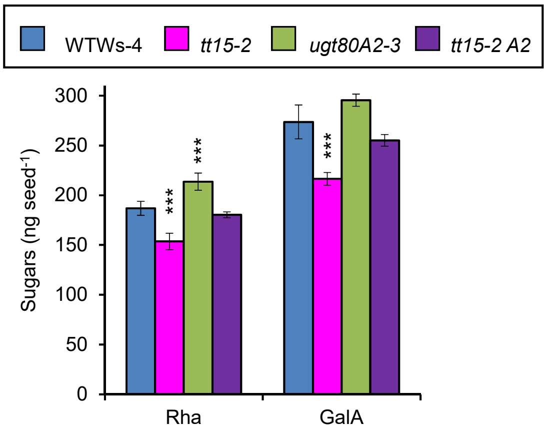

Figure S7. Levels of rhamnogalacturonan I sugars in total mucilage extracted by sonication. Error bars are SE (n=3 to 6). Significant differences from wild type (Kruskal-Wallis test *** $P < 0.001$). Wild type, WT; *tt15-2 ugt80A2-3*, *tt15-2 A2*.

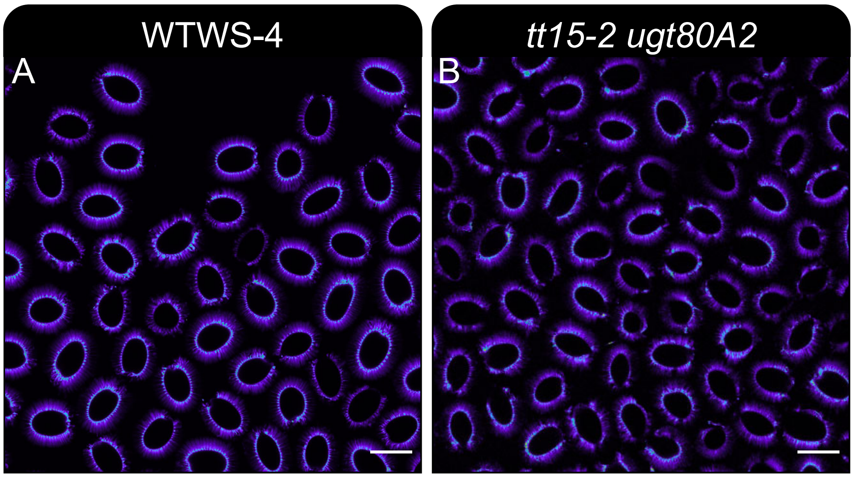

Figure S8. The inner mucilage cellulose of *tt15-2 ugt80A2* seeds has a similar staining intensity with the cellulose specific dye Direct Red 23 to that of wild type (WT) WS-4. Images are shown using the Rainbow2 look-up table. Bars: 500μm.

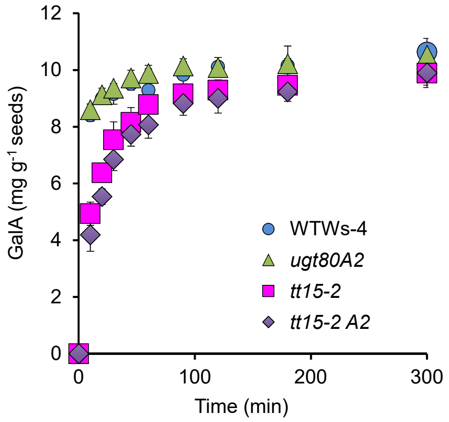

Figure S9. Rate of mucilage release in water expressed as the amount of GalA sugars for a given mass of seed. Error bars represent SD values n=3 biological replicates.

Table S1. Sequences of primers used for genotyping T-DNA insertion for mutant alleles in Ws-4 background.

**Primer**	**5′-to-3′ sequence**
Gene-specific primers	
F-COB16	GAGAGGGATAGTTGATCGAGGTTGG
R-EAL136	TCACACGCCACCACATGGAAGACAAC
R-FCH54	GGAAATCCCAGAATCTGGATC
pGKB5 T-DNA (Versailles collection)	
TAG3 (right border)	CTGATACCAGACGTTGCCCGCATAA

Table S2. Macromolecular characteristics of outer mucilage extracted with water from seeds of wild type, *tt15-2, ugt80A2-3* and *tt15-2 ugt80A2-3*.

	Yield (%)	M_n (kDa)	M_w (kDa)	M_p (kDa)	Rg_n (nm)	$[\eta]_n$ (mL/g)
**Polymeric population 1**						
Wild type	6.5 (0.60)	29380 (1884)	29697 (1853)	31277 (1109)	168 (1.0)	558 (12.1)
tt15-2	5.9 (1.89)	28613 (1625)	28977 (1645)	31837 (1718)	166 (3.2)	544 (3.1)
ugt80A2	7.2 (0.37)	29957 (1364)	30256 (1366)	32887 (1269)	167 (1.6)	554 (1.5)
tt15-2 ugt80A2	6.9 (0.71)	29333 (1746)	29843 (1759)	32883 (1787)	167 (2.5)	534 (5.0)
**Polymeric population 2**						
Wild type	93.5 (0.60)	1114 (34)	1212 (23.2)	995 (35.4)	107 (1.4)	489 (4.0)
tt15-2	94.1 (1.89)	1114 (106)	1201 (122.0)	985 (123.1)	104 (5.1)	485 (8.5)
ugt80A2	92.8 (0.37)	1170 (54.5)	1263 (72.2)	1029 (50.5)	109 (2.5)	485 (2.5)
tt15-2 ugt80A2	93.1 (0.71)	1196 (71.4)	1262 (79.9)	1059 (82.2)	108 (3.2)	480 (2.6)

Values in parentheses are SD of three biological replicates. $M_{n,}$ number-average molar mass; $M_{w,}$ weight-average molar mass; M_{p}, molar mass at peak maximum, Rg_{n}, number average radius of gyration; $[\eta]_{n}$, number-average intrinsic viscosity. All genotypes are in the Ws-4 background.

Table S3. Summary of FITC-dextran molecules used to examine inner mucilage porosity in different genotypes.

**Genotype**	**FITC-dextran size (kD)**			
	**4**	**40**	**70**	**150**
Wild-type	T	T	T	T
tt15-2	T	T	T	T
ugt80A2-3	ND	T	T	T
tt15-2 ugt80A2-3	ND	T	T	T

T, tested; ND, not determined.
